# Impact of the WHO/ICRC Basic Emergency Care (BEC) course on nurses’ knowledge, confidence, and competence in Primary Health Care facilities in Gauteng, South Africa

**DOI:** 10.1016/j.afjem.2025.100890

**Published:** 2025-07-21

**Authors:** Meghan Botes, Lauren Lai King, Robert Holliman, Santel de Lange, Simon Isabwe Tumusiime, Mahlomola Kutoane, Dylan Quiroga, Petra Brysiewicz

**Affiliations:** a Faculty of Health Sciences, Department of Nursing Education, University of the Witwatersrand, Gauteng, South Africa; b Division of Emergency Medicine, Department of Family, Community and Emergency Care, University of Cape Town, Cape Town, South Africa; c African Federation for Emergency Medicine, Cape Town, South Africa; d Stellenbosch University, Department of Nursing and Midwifery, Faculty of Medicine and Health Sciences, Cape Town, South Africa; e National Health Training College, Maseru, Lesotho; f College of Health Sciences, School of Nursing and Public Health, University of KwaZulu-Natal, Durban, South Africa

**Keywords:** Basic Emergency Care (BEC), Primary health care nurses, Emergency care training, Nurse confidence, knowledge improvement

## Abstract

**Introduction:**

Primary health care (PHC) nurses handle preventative care and emergencies, despite the latter not being their focus. Upskilling in basic emergency care is essential as PHC facilities serve as the first point of care. The World Health Organization and International Committee of the Red Cross developed a Basic Emergency Care (BEC) course, but its suitability for Gauteng PHC nursing staff remains unknown. This study assessed the BEC course's impact on nurses' knowledge, confidence, and competence in emergency care at selected Gauteng PHC facilities.

**Methods:**

A pre-post intervention design was used with purposive sampling of eighty-six nurses who completed the BEC course from three emergency departments. Data collection occurred April-June 2024. Knowledge, confidence and competence were assessed using pre- and post-course surveys and knowledge tests, plus a final evaluation with 6 open-ended questions. Data analysis included descriptive statistics, correlational analyses, and inferential methods to determine statistical significance of observed variations.

**Results:**

The study included *N* = 86 nurses across various categories. Knowledge scores significantly increased from 55.1% pre-test to 78.8% post-test. Self-perceived competence and confidence improved from 2.72 to 3.54 post-course. ANOVA revealed significant relationships between nurse categories and scores in pre-tests (*p* = 0.004) and post-tests (*p* < 0.001). Post-test confidence also varied significantly between categories (*p* = 0.046). Content analysis of open-ended responses identified four themes: "Correcting wrongs", "Extremely congested course", "Contextual relevance" and "Skills update."

**Discussion:**

The results highlight significant knowledge gains for all nurses who received BEC training. However, differences in performance between nurse categories suggest a need for further exploration and consideration of how to accommodate different categories of nurses. While nurses found the course highly useful and relevant, content loading and course duration should be considered.

**African relevance**

• Nurses are critical in emergency care and particularly in the nurse led primary health care setting. In Africa, nurses are often the first line providers required to identify and manage acutely ill or injured patients with little formal training in emergency care.
• The Basic Emergency Care (BEC) course was created to train all first-contact healthcare professionals, particularly those without formal training in emergency care, in providing a systematic approach to managing emergency conditions, even in the absence of a diagnosis.
• In resource constrained settings, improving emergency care is identified as a mechanism for reducing mortality and morbidity.

Alt-text: Unlabelled box

## Introduction

Globally, individuals seek emergency care daily for acute life-threatening conditions and injuries [1]. These emergencies include accidents, trauma, severe infections, complications from cardiovascular and chronic illnesses, and maternal health crises. Low- and middle-income countries (LMICs), particularly in Sub-Saharan Africa, bear a disproportionate burden of these emergencies. However, over half of deaths and more than one-third of disabilities in these regions could be prevented with effective emergency care [2]. In South Africa, where 84 % of the population relies on public health care services, Primary Health Care (PHC) facilities often serve as the first point of access [3]. PHC addresses various health needs from prevention to chronic care management [4]. Their accessibility ensures all individuals can access timely healthcare regardless of socioeconomic status [5], emphasizing the need to strengthen PHC, especially emergency care [6].

Emergency services at PHC level address life-threatening conditions and reduce morbidity and mortality [7]. The World Health Organziation (WHO) advocates redirecting efforts toward PHC to achieve Universal Health Coverage, recognising emergency care as essential [8]. WHO resolution WHA 72.16 emphasizes developing these systems for equitable emergency care access [8]. Services in the PHC setting are largely nurse-led and while initially designed for promotive and preventive healthcare, PHC facilities in South Africa have evolved to include emergency care due to their role as the first point of access [9,10].

Nurses play a central role in emergency care teams [11]. In South Africa, nurses fall into distinct categories: professional registered nurses (four years training), enrolled nurses (two years), and auxiliary nurses (one year) [13]. Each category has specific roles and scope of practice within emergency care teams [[12], [14]].

PHC facilities in South Africa are primarily staffed by nurses and a small number of doctors, many of whom have little to no formal emergency care training [15]. Studies in South Africa highlight the need for improved training, particularly for nurses working in primary care settings, improvements in training will equip them with essential emergency care skills and enhance team performance [15,16]. Nurses and doctors in these clinics often work independently under challenging conditions, which can be overwhelming, especially when they lack emergency care, knowledge and skills [17].

Adequate training is crucial for healthcare professionals to deliver quality emergency care. Insufficient training can compromise patient outcomes, leading to delays in recognising urgency, administering treatment, and making referrals [18]. When nurses are adequately trained in basic emergency care, they may feel more confident and less overwhelmed, leading to improved patient outcomes. To address training challenges, the WHO, in collaboration with the International Committee of the Red Cross (ICRC) and the International Federation for Emergency Medicine (IFEM), introduced the Basic Emergency Care (BEC) course in 2018 [19].

The BEC course targets first-contact healthcare professionals, particularly those without formal emergency care training, and provides a systematic approach to managing emergency conditions. It standardises the recognition of patient deterioration and immediate management of emergency conditions [19]. The course employs diverse teaching methods including lectures, facilitator-led discussions, and practical skills stations. In low- and middle-income settings, BEC reportedly increases confidence among participants in providing emergency care [20,21] and is considered applicable to local needs [22]. However, despite these promising outcomes, there remains a lack of research on the specific impact of BEC training on healthcare professionals working in primary healthcare settings.

Recognising the need to expand access to this training, the WHO launched the 25 × 25 Basic Emergency Care campaign in 2023, aiming to train more nurses in 25 countries by 2025 [18]. In alignment with this initiative, training nurses—including professional, enrolled, and auxiliary nurses—in WHO/ICRC BEC equips them with the knowledge and skills to systematically assess patients, initiate key interventions and treatments, and make timely referrals. This study aimed at assessing the impact of the WHO/ICRC BEC course on the nurses’ knowledge, confidence, and competence in providing emergency care in selected PHC facilities in Gauteng province.

## Methods

### Research design

The study employed a pre-post intervention design using a quantitative approach as it was found appropriate for assessing changes attributable to the intervention within the same group over time.

### Research setting

The study was conducted in the Gauteng Province, the most densely populated province in South Africa. The PHC service in Gauteng is organised into various levels of care, ensuring a structured approach to healthcare delivery. These levels include Tertiary, Regional, and District Hospitals, Community Health Centres (CHC), and Primary Health Care Clinics. These are distributed across five regions: City of Johannesburg, City of Tshwane/Metsweding, Ekurhuleni Metro, Sedibeng, and West Rand. Gauteng encompasses a total of 375 Primary Health Care Facilities: 24 Community Health Centres, 332 Primary Health Care Clinics, 6 Community Day Clinics, and 3 Satellite clinics [23]. Primary Health Care Clinics: These clinics represent the foundational level of healthcare. They provide basic services such as vaccinations, maternal and child health, and disease prevention. PHC clinics serve as the first point of contact for the majority of patients. Community Health Centres : CHCs offer a broader range of services than PHC clinics, including specialised care and referrals to higher levels of care. They are equipped to handle more complex medical cases and function as mid-level healthcare facilities [24]. District Hospitals: These hospitals provide a wider range of services than CHCs, including inpatient care, surgery, and specialised services. They serve as the next level of care for patients referred from CHCs or PHC clinics [24]. The three Community Health Care Centres (CHCs) selected for this study were purposely chosen due to their role as mid-level facilities within the PHC service. These centres offer a broad range of services, including 24-hour emergency care, and provide an ideal setting for assessing the impact of emergency care training on nurses working in a dynamic healthcare environment [25].

### Study population

The study population consisted of nurses who had completed the WHO/ICRC BEC course and were practising in PHC facilities in Gauteng. These nurses were purposively selected based on their participation in the BEC training programme and their active roles in the PHC clinics where emergency care is routinely provided.

The WHO/ICRC BEC course was conducted in a blended format, consisting of face-to-face workshops and simulation-based practical sessions, facilitated by the authors who are all certified AFEM BEC trainers using a structured approach aligned with the BEC toolkit. Training sessions were held between 2024 and 2025 across multiple health districts in Gauteng.

The inclusion criteria for participants in WHO/ICRC BEC training were professional Registered Nurse (PN) (3-to-4-year training), Enrolled Nurse (EN) (2-year training), Enrolled Nurse Auxiliary (ENA) (1 year training), Registered or enrolled with the South African Nursing Council, and Dealing with emergencies at the selected facilities.

Nurses were excluded from the study if they: Had not completed the full WHO/ICRC BEC training course, including practical components; Were not actively practicing in a PHC facility at the time of data collection; or Declined to participate or did not provide informed consent.

### Data collection tool

Data was collected using a structured survey including demographic information, knowledge assessment through multiple-choice questions, and self-rating scales for competency and confidence. Competency was measured using Likert-scale questions: Strongly Disagree, Disagree, Agree, and Strongly Agree. Participants rated their confidence and competence in emergency care domains. A final post-course evaluation included 6 open-ended questions.

The BEC course was a four-day face-to-face training programme equipping first-contact healthcare workers with systematic approaches to managing acute illnesses and injuries. The course covered life-saving interventions for trauma, breathing difficulties, shock, and altered mental status through lectures, workbook exercises, skills practice, and case scenarios. Participants were required to attend all sessions and demonstrate skill mastery to receive certification.

### Data collection procedure

Following ethics approval, researchers approached facility managers to establish participant release procedures. Participants volunteered through managers and received information letters. Eight courses ran during April-June 2024. Pre-test data were collected on the first training day before course content delivery. A pre- and post-knowledge test developed and validated by the WHO, ICRC, and IFEM, assessed participants’ learning through 25 scenario-based multiple-choice questions (MCQ) on basic emergency care, requiring a pass mark of 75 % for certification. A course evaluation survey using 6 open ended questions was administered immediately post course.

### Data analysis

Descriptive statistics were used to summarise demographic data and baseline characteristics of participants. Inferential statistics, including correlational analysis and Analysis of Variance (ANOVA), were employed to examine relationships and differences between pre- and post-course assessment scores across different nursing categories (PN, ENA, EN). Open-ended questions from the course evaluation were analysed using quantitative content analysis to identify patterns and categorise participant responses [26].

### Validity and reliability

The study ensured validity through pre- and post-knowledge test standard tools developed by WHO, ICRC, and IFEM. These globally recognised tools align with BEC course learning objectives, ensuring content validity through expert development and international application [27]. Reliability was ensured through consistent administration following standardised protocols, including uniform instructions and digital recording to minimise variations. The structured tools, previously tested across multiple settings, supported internal consistency and reproducibility [28]. Using qualitative content analysis [26], four research team members independently identified meaning units, codes and categories from open-ended questions, then met to discuss, refine and reach consensus.

### Ethical considerations

Ethical approval for the study was obtained from the Human Research Ethics Committee (Medical) of the University of the Witwatersrand (M230835). The permission was sought from the Gauteng Department of Health and the District Research Councils with subsequent permission from facility managers within the provinces for data collection in the various District Health Facilities. Written informed consent was obtained from participants for participation in the study, and anonymity was ensured by using unique codes instead of names, while confidentiality was maintained through secure, password-protected data storage accessible only to the research team.

## Results

### Demographics

A total of 86 nurses completed the BEC course with (*n* = 23) from CHC 1, (*n* = 20) from CHC 2 and (*n* = 43) from CHC 3. We obtained100 % response rate for completion of the questionnaires and open-ended course evaluation Professional categories included 25 % EN, 20 % ENA, and 55 % PN. The data shows that participants had been in their current position for a mean of 7.7 years (SD = 5.5), with both the median (∼6–7 years) and mode (0–10 years) falling within the shortest tenure range and had been qualified for a mean of 10.8 years (SD = 7.8), with the median (∼8–9 years) and mode (0–10 years) both indicating that the majority had relatively recent qualifications despite the wider range of experience levels. (Table 1).

**Table 1:** Study demographics.

Descriptor		Percentage (n)
**Gender**		
Female		97 (*n* = 83)
Male		3 (*n* = 3)
Total		100 (*n* = 86)
**Professional Category**		
Enrolled Nurse		25 (*n* = 22)
Enrolled Nurse Auxiliary		20 (*n* = 17)
Professional Registered Nurse		55 (*n* = 47)
Total		100 (*n* = 86)

Range (years)	Years since Qualified	Years in Current Position
	Percentage ( %)	Percentage ( %)
0–10	57 (*n* = 47)	78 (*n* = 67)
11–20	32 (*n* = 26)	20 (*n* = 17)
21–30	9 (*n* = 7)	1 (*n* = 1)
31+	2 (*n* = 2)	1 (*n* = 1)
Total	100 (*n* = 82) *no response *n* = 4	100 (*n* = 86)

### MCQ knowledge test

**Fig. 1:**
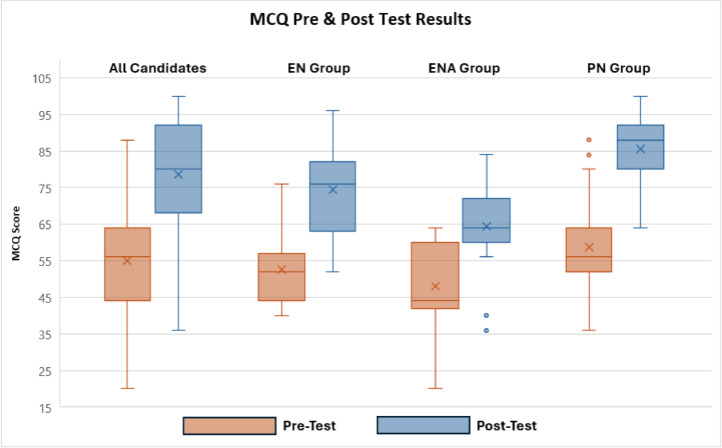
MCQ pre & post test results. *(EN Enrolled Nurse; ENA Enrolled Nursing Auxiliary; PN Professional Registered Nurse)*.

Statistically significant differences were observed between pre- and post-test MCQ scores across all participant groups, with an overall mean test percentage increase from 59 % pre-test to 79 % post-test with the PN’s achieving higher scores. The most notable improvement was also observed in the PN group, where the mean score percentage showed an increase from 59 % pre-test to 86 % post-test (Fig. 1).

ANOVA was conducted to assess variance in scores between the groups (PN, EN & ENA), and effect size analysis was used to measure the magnitude of these differences. The pre-test ANOVA produced an F-statistic of 5.807 and a p-value of 0.004, indicating a statistically significant difference in pre-test scores among the three groups. In the post-test analysis, the F-statistic increased to 23.125, with a p-value of less than 0.001, reflecting an even greater and highly statistically significant variance in post-test scores between the groups (Table 2).

**Table 2:** Pre & post MCQ variance testing.

Measure	Pre-Test Result	Post-Test Result
ANOVA		
**F-statistic**	5.807	23.125
**Significance (p-value)**	0.004	<0.001

Further analysis of scoring differences across the three participant categories was conducted using a Monte Carlo chi-square test with a 95 % confidence interval. A chi-square value of *p* < 0.001 was considered statistically significant. Question-level analysis identified significant pre-test differences between groups in two questions. Post-test results revealed marked improvement in the PN group, with significant differences observed in six questions. The areas where differences were observed related to diagnosis or assessment, medication management, and specific clinical interventions.

The PN group consistently displayed greater improvement in performance than EN and ENA participants in questions involving diagnosis, medication management, and clinical interventions.

### Self-perceived confidence & competence assessment

**Fig. 2:**
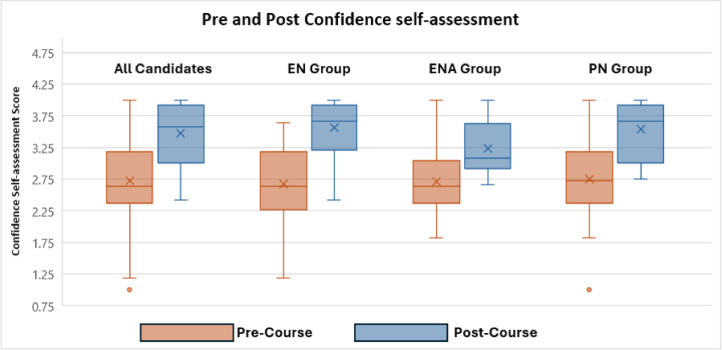
Boxplot score of pre & post confidence self-assessment scores. *(EN Enrolled Nurse; ENA Enrolled Nursing Auxiliary; PN Professional Registered Nurse)*.

The results from self-assessment on participants’ confidence showed an increase in mean confidence scores for managing emergencies across all categories of nurses, rising from 2.72 pre-course to 3.54 post-course on a four-point Likert scale (Fig. 2).

High internal consistency was found with a Cronbach alpha of 0.929 for pre-test results and 0.933 for post-test results. ANOVA revealed a statistically significant change in self-perceived confidence variation between categories post-course (*p* = 0.046), while no significant difference was noted in the pre-course (*p* = 0.889).

The mean score for self-perceived competence in all categories of nurses’ pre-course leaned toward the mid to upper end of the scale. Self-perceived competence scores did not change significantly from pre to post course scores.

### Open-ended course evaluation findings

Written narratives were used to collect data on the participants' post course evaluation. Using content analysis four categories emerged namely, “Correcting wrongs”, “Extremely congested course”, “Contextual relevance and Skills update,”

#### Correcting wrongs

The participants described how the course showed them “*how to do things properly*” – that they gained the appropriate knowledge and could approach a patient with confidence and without fear, knowing that what they were doing to care for the patient was correct. Many of the participants mentioned that the course “*has changed my daily practice*” and “*corrected most wrong practices*”.

The participants explained that the training showed them that there was a particular order to follow in managing such patients. In following this approach, they had become confident in their own knowledge and could carry out the nursing management “*without fear or doubt*”. A participant described how this then meant that she now knew what to do in emergency care and expressed confidence along with other participants who echoed *“Now I will be able to make my own clinical assessment and manage appropriately”*

#### Contextual relevance

Many did describe the course to be very exciting and relevant; *“whatever we are learning from this course is what we are facing at the workplace”,* and that the course should be for *“every facility in the country”*. Participants also expressed the need for all members of the multidisciplinary team to receive training, *“Can we please have doctors participate as well”, “All my co-workers should attend this course to make our work easier and our patients' lives will be saved”*. One participant suggested that the course be tailored to suit the different categories’ scope of practice *“As an ENA, I would change to focus only on my scope of practice”.*

#### Skills update

Participants were enthusiastic about new skills learned as well as skills updates and refresher training *“I have gained a lot of skills that I didn’t have before”.* One participant expressed a broadening of her knowledge on bleeding control *“In most cases I used to do direct pressure, not knowing that there are other dressings that are used to stop bleeding”,* referring to the various skills taught to arrest bleeding. Participants also expressed the anticipated improvement in triage processes in their clinical areas saying *“Triage will be better” and “They help us identify patients that are at risk”*

#### Extremely congested course

Many of the participants spoke about the fact that the course covered a great deal of content that felt squashed into a small period of time. Many of the participants described how they would appreciate reading material prior to the course, thus having more time to assimilate the theoretical content. Participants needed *“time to acclimatise”* and noted how some suffered from “*brain strain*” after listening for 8 h. Many described how they would like to return for an update or refresher course in the future.

## Discussion

This study represents the first evaluation of WHO/ICRC BEC training for nurses in primary healthcare settings across different nursing categories. The discussion focuses on three domains: knowledge, confidence, and self-perceived competence before and after BEC completion.

Participants demonstrated significant post-course knowledge improvements across all nursing categories, with greatest gains among PNs, particularly in diagnosis, medication management, and clinical interventions. These findings align with previous BEC evaluations [10,22,29]. However, more modest gains among ENs and ENAs suggest the assessment content may align more closely with PN scope of practice and baseline training. Similar patterns were described by Olufadeji et al. [30], who found limited improvement among non-physician participants in specific question types. This divergence likely reflects variations in clinical responsibility and emergency protocol familiarity rather than course limitations. These findings reinforce BEC's value as foundational emergency care training while highlighting needs for nuanced evaluation tools.

Participants reported marked confidence increases when managing emergency conditions. This addresses lack of confidence as a barrier to effective emergency care among nurses in primary healthcare settings [31–33]. Increased confidence enables improved clinical performance in high-stakes situations [34]. The confidence shift occurred across all nursing categories, including those with modest knowledge gains. This increase in self-assurance is particularly relevant given the decentralised nature of emergency care delivery in many low-resource contexts, where nurses often serve as the first point of contact [35].

While participants reported marked improvements in knowledge and confidence, self-perceived competence showed limited change following the course. This finding aligns with other studies indicating that increases in confidence do not necessarily translate into an immediate sense of clinical competence, particularly after short-format training [31,32]. It is likely that competence, unlike confidence, is more closely tied to repeated practice and supervised clinical exposure—elements not directly addressed through a brief training intervention. Despite this, participants strongly endorsed the course's relevance to their clinical responsibilities and highlighted its value in correcting misconceptions and refining their approach to emergency care. This perceived relevance is a key factor in adult learning, with literature showing that educational interventions are more likely to be effective when learners recognise their direct applicability to practice [36,37]. In our study, participants described the course as equipping them with a structured and sequential method for patient assessment, which they viewed as a meaningful shift from previous, more fragmented approaches. These findings suggest that although participants may not yet feel fully competent, the course contributed to an important transitional stage, strengthening foundational understanding and promoting the adoption of a more systematic clinical approach. This trajectory aligns with the broader understanding that competence evolves through cumulative experience, reflection, and reinforcement [38]. Future evaluations may benefit from incorporating additional measures of clinical behaviour or simulated performance to more accurately assess competence, particularly in cadres with limited prior emergency care exposure. Doing so would help ensure that training outcomes are not judged solely by immediate self-perception, but also by demonstrated application in practice.

In addition to the core domains of knowledge, confidence, and competence, several other important themes emerged from participants’ feedback regarding the structure, delivery, and long-term relevance of the course. Participants highlighted the course’s role in correcting incorrect practices and introducing a structured approach to emergency care. Many described feeling empowered to manage patients more confidently, not only because of specific knowledge gained but due to the adoption of a systematic and sequential method of assessment and intervention. This shift from fragmented to integrated care was seen as a critical learning outcome and reflects one of the BEC course’s core strengths; its emphasis on a unified approach to managing acutely ill or injured patients [39]. However, despite these perceived benefits, participants consistently reported that the course content felt overly dense. The intensity and pace left limited room for reflection or consolidation, particularly among those with less formal exposure to emergency care principles. Suggestions for extended course durations, pre-course reading material, and regular refresher training reflect a broader need to optimise delivery for sustained learning and retention [40]. This feedback is particularly relevant in settings where clinical duties and staffing constraints limit extended off-site training feasibility. The call for ongoing educational support underscores that emergency care competence requires continuous reinforcement. This aligns with findings by Michaeli et al. (2023) [20], who noted that in low-resource environments, isolated training events are insufficient to maintain proficiency. Instead, layered learning models incorporating refresher training, mentorship, and embedded clinical reinforcement may offer more sustainable long-term capacity building strategies.

## Limitations

The research was conducted in three facilities within one province in South Africa, limiting its generalisability to other primary healthcare services beyond the study context. The sample size may not fully represent the broader nursing population, and self-reported data on confidence, and self-perceived competence could introduce biases. This was mitigated through the use of anonymous data collection, clear instructions emphasising the importance of honest responses, and the use of validated assessment tools. The short-term nature of the assessment does not provide insight into the sustainability of the intervention’s impact. Despite these limitations, the study offers valuable insights into the effectiveness of structured educational interventions for nurses providing emergency care in primary healthcare settings, while highlighting areas for future research including longer-term follow-up studies and multi-site evaluations.

## Conclusion

The WHO/ICRC BEC training approach is beneficial for knowledge gain, improvement in confidence in emergency care skills and is relevant to nurses practicing in primary health care settings in Gauteng, South Africa. While the course aims to serve diverse healthcare professionals, conventional knowledge scores may not fully capture it's impact across different nursing units. The varying knowledge score improvements observed in this study reflect the inherent diversity in participants' professional backgrounds, scopes of practice, and baseline expertise. Each nursing category brings unique training foundations and operates within distinct regulatory boundaries that shape how they can apply emergency care principles in practice. The enthusiastic narrative evaluations from participants across all professional levels suggests that meaningful learning occurred beyond what standardized metrics could measure. These qualitative insights reveal the course's practical value in ways that quantitative assessments alone cannot detect, highlighting the importance of multidimensional evaluation approaches that consider each unit’s specific clinical context and authorised responsibilities when determining training impact. The study highlights the opportunity to explore various assessment methods to cater for different cadres of nurses and other health care professionals. There is also a need to explore qualitative evaluation of educational interventions such as the WHO/ICRC BEC course.

## Dissemination of results

Results from this study have been to all data collection sites. Findings have been presented at two local research forums (one of which including some participants of the study) and one international conference where the abstract of the presentation is published.

## Author contribution

M.B., L.L., P.B. conceptualization, project administration, implementation, investigation, writing - original draft M.B., D.Q., data curation and validation M.B., R.H., L.L., P.B., writing - review & editing - all authors supervision P.B.

## Ethical statement

Ethical approval for the study was obtained from the Human Research Ethics Committee (Medical) of the University of the Witwatersrand and permission was sought from the Gauteng Department of Health and the District Research Councils with subsequent permission from facility managers within the provinces for data collection in the various District Health Facilities (M230835) Written informed consent was obtained from participants for participation in the study.

## Funding source

This research was supported by the University of the Witwatersrand through the Female Academic Leadership Fellowship and the National Research Foundation Thuthuka grant. The funding was utilized for operational aspects of the project, including costs associated with training facilitators, conducting courses, and providing a stipend for a research assistant.

## Declaration of competing interest

The authors declare that they have no known competing financial interests or personal relationships that could have appeared to influence the work reported in this paper.
